# The His131Arg substitution in the FCGR2A gene (rs1801274) is not associated with the severity of influenza A(H1N1)pdm09 infection

**DOI:** 10.1186/s13104-016-2096-1

**Published:** 2016-06-07

**Authors:** Alvino Maestri, Vinicius Albuquerque Sortica, Deimy Lima Ferreira, Jessylene de Almeida Ferreira, Marcos Antônio Trindade Amador, Wyller Alencar de Mello, Sidney Emanuel Batista Santos, Rita Catarina Medeiros Sousa

**Affiliations:** Alvino Maestri Neto, Laboratório de Genética Humana e Médica, Universidade Federal do Pará, Cidade Universitária Prof. José da Silveira Neto, Rua Augusto Corrêa, 01, BOX 8615, CEP 66.075-970 Belém, Pará Brazil; Núcleo de Pesquisas em Oncologia, Universidade Federal do Pará, Belém, Pará Brazil; Laboratório de Vírus Respiratórios, Seção de Virologia Instituto Evandro Chagas, Ananindeua, Pará Brazil; Laboratório de Genética Humana e Médica, Universidade Federal do Pará, Belém, Pará Brazil; Instituto Evandro Chagas, Universidade Federal do Pará, Belém, Pará Brazil

**Keywords:** A(H1N1)pdm09 infection, Influenza, FCGR2A

## Abstract

**Background:**

The virulence and pathogenicity of different influenza strains are responsible for a more or less severe disease. Recent studies have attempted to understand how host genetic factors may influence the clinical presentation of the disease. In the present study, the His131Arg (rs1801274) polymorphism was investigated in individuals from a Brazilian admixed population with a diagnosis of influenza A(H1N1)pdm09 infection.

**Methods:**

In the present study, the influence of the His131Arg (rs1801274) polymorphism, a variant of the FCGR2A gene, was investigated in 436 patients with a diagnosis of influenza A(H1N1)pdm09, evaluated at health services in the northern and northeastern regions of Brazil between June 2009 and August 2010. Patients were divided into a group of non-hospitalized patients (n = 192) and a group of hospitalized patients (n = 244; 100 of them died).

**Results:**

No significant difference in the allele or genotype frequencies of the rs1801274 polymorphism was observed between groups (p = 0.952 and p = 0.388). Multinomial logistic regression showed no effect of the rs1801274 polymorphism on severity or death of patients from the Brazilian admixed population (p = 0.368 and p = 0.469).

**Conclusions:**

The rs1801274 polymorphism is not associated with severe disease in patients infected with influenza A(H1N1)pdm09.

## Background

Influenza, or the flu, is an acute respiratory tract infection caused by the influenza virus, which shows a global distribution and high transmissibility. In April 2009, the Centers for Disease Control and Prevention (CDC) reported two cases of influenza in children from California, USA [1], caused by a new influenza strain. This strain originated from the genetic rearrangement of four other circulating viruses and resulted in the first influenza pandemic in the 21st century [2]. The increase in severe cases and deaths among children and young adults called attention during the pandemic period [3], without the death rate exceeding annual figures [4]. The estimated rate of deaths caused by the 2009 pandemic influenza A(H1N1)pdm09 worldwide was 65 % in individuals aged 18–64 years [5]. In contrast, in southern Brazil, the median annual rate of underlying respiratory/circulatory deaths was 70 % in patients aged ≥60 years between 2000 and 2008 [6].

Although the virulence and pathogenicity of different viral strains are responsible for a more or less severe disease, recent studies have attempted to understand how host genetic factors may influence the clinical presentation of the disease, considering signaling pathways activated by viral infection [7], as well as pathways activated by the immune system in an attempt to promote viral clearance [8]. Recent studies have tried to identify host genetic variants that could explain severe cases of the disease: TNF [9], IFTM3 [10], CCR5 [11, 12], and ST3GAL1 [13].

In 2012, a case–control study was published in which 91 patients who developed severe pneumonia caused by influenza A(H1N1)pdm09 were compared to 98 asymptomatic household contacts. The results showed that 36.6 % of the patients with severe pneumonia were homozygous for allele A of the rs1801274 variant of the FCGR2A gene, which encodes a histidine (His) at position 131 of the protein, while only 13.2 % of the contacts who did not develop the disease carried this variant. The authors concluded that this variant of the FCGR2A gene may be related to greater susceptibility to severe forms of the disease [14].

FCGR is a glycoprotein that binds to the Fc region of immunoglobulin G, triggering immune responses such as pathogen phagocytosis, clearance of immune complexes, antigen presentation, and degranulation [15]. Five genes are found on chromosome 1 (FCGR2A, FCGR2B, FCGR2C, FCGR3A, and FCGR3B), which encode five receptors with low affinity for this glycoprotein. The activation or inhibition of these receptors determines the local inflammatory response [16]. The genetic variant of the IgG Fc receptor at position 131 has been associated with susceptibility to inflammatory and infectious diseases [17–20]. In the present study, the His131Arg (rs1801274) polymorphism was investigated in individuals from a Brazilian admixed population with a diagnosis of influenza A(H1N1)pdm09 infection.

## Methods

In the present study, the His131Arg substitution in the FCGR2A gene (rs1801274) was studied in nasopharyngeal swab samples obtained from 436 subjects that were randomly selected among 1524 cases from the northern and northeastern regions of Brazil diagnosed with influenza caused by strain A(H1N1)pdm09 between June 2009 and August 2010. Collection of the material during the study was accompanied by filling out a notification form of the Brazilian National System of Medical Care (SINAM), which contained the clinical data of the patient.

Viral detection was carried out at the Laboratory of Respiratory Viruses, virology section of the Evandro Chagas Institute (Seção de Virologia do Instituto Evandro Chagas—SEVIR/IEC), Ananindeua, Pará, Brazil, using the SuperScript III™ One-Step RT-qPCR System with Platinum^®^ Taq (Invitrogen Life Technologies). Using the same material, genomic DNA was extracted from patient samples with the QIAamp DNA Mini Kit (Qiagen) according to manufacturer instructions. The extracted DNA was quantified in a NanoDrop spectrophotometer (Uniscience^®^) at 260 to 280 nm and submitted to real-time PCR using the C__9077561_20 TaqMan^®^ assay (Applied Biosystems) according to manufacturer instructions. The proportions of African, European and Amerindian genetic ancestry of the patients were estimated using a panel of 48 ancestry informative markers as described elsewhere [21].

The allele and genotype frequencies of the polymorphism studied were estimated by direct counting. The individual proportions of genetic ancestry were estimated using the STRUCTURE 2.3.3 software [22]. Differences in the main characteristics between the groups of patients were verified using the Student *t* test, Fisher’s exact test and Kruskal–Wallis test. Fisher’s exact test was also applied to evaluate differences in the allele and genotype frequencies of the His131Arg (rs1801274) polymorphism between the groups of patients. Multinomial logistic regression controlling for age, comorbidities and European and African ancestry was performed to evaluate the existence of an association between the polymorphism and severity of infection. Statistical analysis was performed using the SPSS18.0 software, adopting a level of significance of p < 0.05.

### Ethics approval and consent to participate

All patients enrolled in the study provided their written informed consent. The study was approved by the Ethics Committee of the Center of Tropical Medicine, Federal University of Pará (Núcleo de Medicina Tropical, Universidade Federal do Pará).

## Results

The sample of 436 subjects with the disease was divided into two groups (Table 1) according to disease progression: a group of non-hospitalized patients (n = 192) and a group of hospitalized patients (n = 244; 100 of them died). The characteristics of the subjects are shown in Table 1. There was a predominance of women (62.8 %, n = 247), with a mean age of 24.6 years. Of these (n = 247), 193 were of child-bearing age, 63 were pregnant, and 40 required hospitalization (22 died). The absence of comorbidities in non-hospitalized patients was significant (p = 0.007). Among patients with comorbidities (n = 150), metabolic disorders (p = 0.001), immunosuppression (p < 0.001) and obesity (p = 0.001) were the most frequent in severe cases of the disease.

**Table 1:** Clinical characteristics and genotypes of patients infected with influenza A(H1N1)pmd09 virus

Characteristics	All patients	Non-hospitalized	Hospitalized		p
			Survived	Died	
N	436	192	144	100	
Female gender	274 (62.8)	116 (60.4)	92 (63.9)	66 (66.0)	0.616^a^
Age (year)	24.6 ± 15.6	23.6 ± 14.3	21.4 ± 15.0	30.9 ± 17.1	<*0.001* ^b^
Pregnant	63 (32.1)	23 (28.4)	18 (28.1)	22 (43.1)	0.157^a^
Smoking	23 (5.3)	7 (3.6)	6 (4.2)	10 (10.0)	0.081^a^
Abnormal chest radiograph	135 (31)	0 (0)	70 (48.6)	65 (65.0)	<*0.001* ^a^
Without comorbidities	286 (65.6)	52 (27.1)	53 (36.8)	45 (45.0)	*0.007* ^a^
With comorbidities					
Chronic lung disorder	86 (19.7)	33 (17.2)	36 (25.0)	17 (17.0)	0.169^a^
Chronic cardiovascular condition	32 (7.3)	10 (5.2)	10 (6.9)	12 (12.0)	0.110^a^
Metabolic disorder	19 (4.4)	2 (1.0)	6 (4.2)	11 (11.0)	*0.001* ^a^
Immunosuppression	8 (1.8)	0 (0)	1 (0.7)	7 (7.0)	<*0.001* ^a^
Obesity	9 (2.1)	1 (0.5)	1 (0.7)	7 (7.0)	0.001^a^
Hemoglobinopathy	3 (0.7)	1 (0.5)	1 (0.7)	1 (1.0)	0.895^a^
Chronic kidney disease	4 (0.9)	1 (0.5)	2 (1.4)	1 (1.0)	0.822^a^
Genetic ancestry					
Native American	0.201 (0.029;0.932)	0.186 (0.022;0.610)	0.219 (0.041;0.821)	0.213 (0.035;0.906)	0.345^c^
European	0.613 (0.046;0.930)	0.638 (0.046;0.919)	0.585 (0.095;0.928)	0.578 (0.059;0.930)	*0.026* ^c^
African	0.132 (0.022;0.676)	0.125 (0.022;0.610)	0.138 (0.30;0.676)	0.149 (0.024;0.504)	*0.036* ^c^
FCGR2A His131Arg (rs1801274)					
AA	157 (36.0)	73 (38.0)	47 (32.6)	37 (37.0)	
AG	195 (44.7)	80 (41.7)	74 (51.4)	41 (41.0)	0.388^a^
GG	84 (19.3)	39 (20.3)	23 (16.0)	22 (22.0)	
A^d^	509 (58.4)	226 (58.9)	168 (58.3)	115 (57.5)	0.952^a^
G^e^	363 (41.6)	158 (41.1)	120 (41.7)	85 (42.5)	

Age is reported as the mean ± SD, genetic ancestry is reported as the median (minimum; maximum), and all other variables are reported as absolute number (%)

^a^Fisher’s exact test

^b^Student t-test

^c^Kruskal–Wallis test

^d^Adenine

^e^Guanine

The study of genetic ancestry in the population studied showed a median European contribution of 61.3 %, an Amerindian contribution of 20.1 %, and an African contribution of 13.2 %. The frequency of the homozygous AA genotype of the His131Arg polymorphism was 38 % in non-hospitalized patients, 32.6 % in hospitalized patients who survived, and 37 % in hospitalized patients who died, with no significant difference among groups (p = 0.388). Multinomial logistic regression controlling for European and African genetic ancestry, comorbidities and age revealed no significant association between the FCGR2A genotypes and severity of the disease (hospitalization) or death of the patients (p = 0.368 and p = 0.469, respectively).

## Discussion

A case–control study followed by a meta-analysis demonstrated an increased risk of developing Kawasaki disease in carriers of allele A in the Chinese Han population [17]. In another meta-analysis, the His131Arg polymorphism in the FCGR2A gene was found to be associated with susceptibility to systemic lupus erythematosus and lupus nephritis in Asian populations [18]. This polymorphism was also associated with bacteremia and the severity of pneumonia in patients hospitalized in Spain [19]. In contrast, in intensive care unit patients with a diagnosis of invasive *Streptococcus pneumoniae* infection (202 with pneumonia and 55 with meningitis), the GG genotype (guanine) of the FCGR2A His131Arg polymorphism exerted a protective effect (OR 0.32) [20].

Although allele A of the FCGR2A gene (rs1801274) has recently been associated with greater susceptibility to severe forms of infection with influenza A(H1N1)pdm09 in the Mexican population in which the Amerindian contribution is high (approximately 60 %) [14], the present study found no association of this polymorphism with more severe influenza infection in the Brazilian admixed population. These discordant results may be attributed to differences in the genetic composition of the populations studied.

## Conclusions

The present study included a large sample and the symptoms of the patients were rigorously characterized, facts that support the results found. Some studies have reported an association between the His131Arg (rs1801274) polymorphism in the FCGRA gene and more severe influenza infection. Our findings demonstrate that the polymorphism is not associated with an unfavorable outcome in patients infected with influenza A(H1N1)pdm09 in the Brazilian admixed population. Further studies are needed to better understand the effects of host genetic variants on the susceptibility to and severity of infections caused by influenza A(H1N1)pdm09.

## Abbreviations

CDC: Centers for Disease Control and Prevention
IEC: Evandro Chagas Institute
SEVIR: virology section
WHO: World Health Organization
A: adenine
G: guanine
